# Outcomes with intracytoplasmic sperm injection of cryopreserved sperm from men with spinal cord injury

**DOI:** 10.1186/2051-4190-23-14

**Published:** 2013-12-02

**Authors:** Shaliha Bechoua, Yasmine Berki-Morin, Frédéric Michel, Sophie Girod, Paul Sagot, Patricia Fauque

**Affiliations:** Hôpital de Dijon, Laboratoire de Biologie de la Reproduction, Université de Bourgogne, 21079 Dijon, France; Hôpital de Dijon, Service de Chirurgie Urologique-Andrologie, Université de Bourgogne, 21079 Dijon, France; Hôpital de Dijon, Université de Bourgogne, Service de Gynécologie-Obstétrique, Médecine Fœtale et Stérilité Conjugale, 21079 Dijon, France

**Keywords:** Fertility, Spinal cord injury, Pregnancy outcomes, Cryopreservation, Assisted reproductive technology, Assistance Médicale à la Procréation, Congélation de sperme, Infertilité, Lésion médullaire

## Abstract

**Background:**

Erectile dysfunction, ejaculatory dysfunction and poor semen quality are the main causes of infertility in men with spinal cord injury (SCI). Different sperm retrieval techniques such as penile vibratory stimulation (PVS), electro-ejaculation (EEJ) or surgical sperm retrieval (SSR) associated or not with sperm cryopreservation can be offered to these patients to preserve their fertility. If fatherhood cannot be achieved naturally, assisted reproductive techniques can be offered to these patients using either fresh or frozen/thawed sperm. The aim of the study was to report in SCI patients from Dijon (Burgundy) and in the literature, intracytoplasmic sperm injection (ICSI) outcomes using frozen sperm obtained either by antegrade ejaculation (PVS or sexual intercourse) or by SSR.

**Methods:**

A retrospective analysis was performed in Dijon, Burgundy over a 17 year period (1995-2011) on a cohort of 19 SCI men (6 quadriplegics and 13 paraplegics, mean age: 25.2 ± 5.6 years) who underwent a sperm cryopreservation followed or not by intracytoplasmic sperm injection (ICSI). Patients were divided into two groups according to the sperm retrieval method used (antegrade ejaculation group (n=10): penile vibratory stimulation (PVS) for 9 patients and sexual intercourse for 1 patient and surgical sperm retrieval (SSR) group: n=9). The sperm parameters in both groups were analyzed. Pregnancy outcomes in the 8 couples who underwent ICSI were evaluated.

**Results:**

The fertilization rates were 57 and 55%, the embryo’s cleavage rates were 90 and 93% in the antegrade ejaculation and SSR groups respectively. Among the 8 couples who underwent ICSI, 5 couples achieved pregnancy. The pregnancy rates per couple were 50% and 75% in the antegrade and SSR groups respectively.

**Conclusions:**

Although some studies don’t recommend freezing sperm in SCI patients, the pregnancy rates presented in this study are encouraging and warrant the use of frozen/thawed sperm in very specific situations.

## Résumé

**Introduction:**

L’infertilité chez l’homme blessé médullaire s’explique d’une part par une dysfonction érectile et éjaculatoire et d’autre part par l’altération de la qualité des paramètres spermatiques. Différentes techniques facilitant le recueil de sperme (vibromassage, électro-éjaculation et prélèvement chirurgical de spermatozoïdes) peuvent être proposées à ces patients. Le recueil de sperme peut s’accompagner ou non d’une autoconservation. Si une grossesse n’est pas obtenue naturellement alors des techniques d’assistance médicale à la procréation peuvent être envisagées (insémination, fécondation *in vitro* avec ou sans micro-injection). Le but de ce travail a consisté à évaluer les issues de grossesses dans la littérature et sur une cohorte de patients de Dijon, blessés médullaires ayant eu recours à de la fécondation *in vitro* avec micro-injection (ICSI) à partir de spermatozoïdes cryoconservés éjaculés ou chirurgicaux.

**Méthodes:**

Il s’agit d’une étude rétrospective réalisée sur la base de registres des patients pris en charge au CECOS (centre d’étude de la conservation des œufs et du sperme) de Dijon entre 1995 et 2011 (6 patients tétraplégiques, 13 patients paraplégiques, moyenne d’âge : 25.2 ± 5.6 ans). Pour ces 19 patients, une autoconservation de sperme a été réalisée et 8 de ces patients ont eu recours à l’ICSI. Deux groupes de patients ont été définis, le groupe pour lequel les spermatozoïdes ont été obtenus par éjaculation antégrade (n=10 : 9 patients par vibromassage et 1 après rapports sexuels) et le groupe pour lequel les spermatozoïdes ont été obtenus chirurgicalement (n=9). Dans les deux groupes, les paramètres spermatiques ont été analysés ainsi que les issues de grossesses obtenues par ICSI réalisées avec des spermatozoïdes congelés.

**Résultats:**

Dans les groupes «éjaculation antégrade » et « prélèvement chirurgical », les taux moyens de fécondation par micro-injection étaient respectivement de 57% et 55% et les taux moyens de clivage embryonnaire de 90% et 93%. Parmi ces 8 couples, 5 ont obtenu une grossesse. Les taux de grossesse par couple étaient de 50% dans le groupe «éjaculation antégrade » et de 75% dans le groupe « prélèvements chirurgicaux ».

**Conclusions:**

Bien que certaines équipes ne recommandent pas de réaliser une autoconservation de sperme chez les patients blessés médullaires, cette possibilité apparait selon les résultats présentés dans cette étude comme une option préventive satisfaisante.

## Background

A thousand new cases of spinal cord injury occur annually in France. The annual incidence of people with traumatic spinal cord injury (SCI) in Burgundy (France) was in 2000, 18 persons (75% being men) per 1,610.000 inhabitants [1, 2]. Overall, most SCI patients are young men, 52% of them being less than 35 years old (27% between the age of 18 and 25 years old and 25% between the age of 26 and 35 years old) (data from the Irme: Institut pour la recherche sur la moelle épinière et l’encéphale : http://irme.org). Most of the SCI men did not achieve parenthood. Indeed, in France, the mean age for the first child was 31 years old (for men born in 1970) [3].

It is well known that following SCI, most men experience fertility-related problems because of impairments in erectile and ejaculatory functions and because of poor semen parameters. Hence, these men require medically assisted ejaculation (penile vibratory stimulation: PVS and electroejaculation: EEJ) in order to obtain a semen sample. In case of PVS and EEJ failure, surgical sperm extraction (SSR) can be performed.

Numerous studies have focused on characterizing semen quality in SCI patients [4–7]. These studies indicated a decrease in both the number of motile spermatozoa and the number of viable spermatozoa but a normal sperm count. The causes of poor semen quality (motility and viability) in men with SCI are not fully understood. Lack of ejaculation, testicular hyperthermia, genito-urinary tract infections do not seem to account for the poor semen quality [4]. However, it has been published that low sperm motility could be due to factors present in the seminal plasma. For instance, elevated concentrations of white blood cells and activated T-cell subpopulations [8] and elevated concentrations of cytotoxic cytokines were found in the seminal plasma of these men [9]. Brackett et al. [10] demonstrated that interference with the activity of IL-6, IL-1β, and TNF-α at the receptor level improved sperm motility in men with SCI whose semen had elevated concentrations of these cytokines. In addition, cytokines present in seminal plasma increase the production and release of reactive oxygen species, reducing both sperm motility and the ability of the spermatozoa to fuse with the oocyte [11].

In SCI patients, retrospective studies have shown that semen parameters do not significantly decline post-injury [12, 13], which led to the conclusion that cryopreservation is not an option for fertility preservation in patients with spinal cord injury. In addition, it has been demonstrated that cryopreservation induces a decrease in sperm motility and mitochondrial activity while increasing DNA fragmentation [7]. It is worth pointing out that these alterations are not restricted to the sperm of SCI patients but can be observed after cryopreservation of sperm in non-SCI patients. Nevertheless, the only way to address the semen alteration issue and to rule out cryopreservation as a forthcoming option for preservation of fertility would be to design a prospective study aiming at evaluating the evolution of semen parameters along with DNA fragmentation and oxidative stress over time. So far, this type of study has never been undertaken. Until then, sperm cryopreservation should be considered as it remains a simple, safe and affordable preventative procedure for fertility management [14]. For SCI patients who wish to use their cryopreserved sperm to achieve parenthood, intracytoplasmic sperm injection is often the treatment used because of poor semen quality after thawing [7].

However, regarding ICSI outcomes using frozen/thawed sperm obtained by PVS or SSR in SCI patients, only few studies have been published in the literature. Indeed, in the past 10 years, 8 studies have focused on the ICSI outcomes using spermatozoa obtained by SSR and PVS. For ICSI outcomes using spermatozoa obtained by SSR, a total of 7 studies were published with only one reporting ICSI outcomes using frozen/thawed spermatozoa [15]. In this study, only one patient underwent ICSI with frozen/thawed sperm obtained from vasal aspiration. The case report by Kyono et al. [16] was carried out with fresh spermatozoa obtained by SSR. As for the study undertaken by Mc Guire [17], 3 patients underwent testicular biopsy. However, in this study, it is difficult to find out if these patients underwent ICSI. In the study by Kanto et al. [18], testicular sperm was retrieved in 19 cases out of the 22 men with SCI. Fourteen patients underwent ICSI with fresh sperm and 4 with frozen/thawed sperm. However, in the study by Kathiresan et al.[19], ICSI was performed with fresh and frozen/thawed sperm without knowing when fresh sperm vs frozen/thawed sperm was used. Finally, in 2 studies, the authors did not indicate whether the spermatozoa used for ICSI were fresh or frozen/thawed [20, 21].

As far as the ICSI outcomes using spermatozoa obtained after PVS are concerned, only 2 studies have been published [19, 22]. The first one published in 2001 by Pryor et al. [22] used ICSI as the last option when IUI failed (4 out of 11 patients). However, in this study 2 out the 11 patients underwent PVS and it is not clear whether or not these patients underwent ICSI. Finally, in the study by Kathiresan et al. [19] reporting ICSI outcomes after PVS in 12 patients, the authors did not discriminate between fresh vs frozen/thawed sperm.

Overall, the literature lacks publications regarding ICSI outcomes using cryopreserved sperm obtained by either PVS or SSR in SCI patients. Therefore, the aim of the present study was twofold: i) to investigate the results of ICSI outcomes using frozen/thawed sperm obtained after PVS or SSR and ii) to compare our results with the ones published in the literature.

## Methods

### Patients

A retrospective analysis was performed on a cohort of 19 men with SCI who underwent sperm cryopreservation in the CECOS (Centre d’Etudes de la Conservation des OEufs et du Sperme: Egg, Sperm Embryo banking) of Dijon’s Hospital from 1995 to 2011. Spermatozoa were retrieved using either penile vibration stimulation (PVS, n=9), surgical sperm extraction (SSR, n=9) or after sexual intercourse (SI, n=1). Two groups were defined, the antegrade ejaculation group (PVS and SI, n=10), the SSR group (testicular sperm extraction: TESE and microsurgical epididymal sperm aspiration: MESA). From both groups, a total of 8 couples seeking infertility treatment underwent ICSI procedure (Intra Cytoplasmic Sperm Injection). Female partners in both groups underwent a basic infertility evaluation by a gynaecologist.

### Patients’ follow-up

The 11 patients who did not undergo ART (6 and 5 in the antegrade and surgical groups respectively) were contacted by telephone. Nine of these patients were single at the time of the accident and 2 were married with children. Two patients out of 11 were lost to follow-up. The patients were asked the following questions.

For the singles at the time of the accident: are you still single? If not, are you planning on having a baby? Does having frozen sperm make you feel more secure about your fertility and your future parenthood?

For the patients married at the time of the accident: are you still married? Did you have another baby or are you planning on having another baby?

### Sperm retrieval methods

#### Sexual intercourse

For one of the patients, sperm was retrieved and frozen after sexual intercourse using non-spermicidal condoms (Male factorPak, Inhealth, Dublin, Ireland). Sperm was collected and allowed to liquefy for 30 min at room temperature (RT). Then, sperm parameters were determined and the freezing performed (see procedures below).

#### Penile vibratory stimulation

PVS has been recommended as the first line of treatment for anejaculation in men with SCI. Therefore, PVS (Ferticare®, Multicept, Albertslund, Denmark) was performed on all patients except for the patient who could ejaculate via sexual intercourse. PVS was performed by placing the vibrating device on the dorsum or frenulum on the glans penis until antegrade ejaculation occurred with a maximum of 10 min. The amplitude and the frequency used were 2.5 mm and 100 Hz respectively [23].

For 9 patients, semen samples after PVS were collected. Four out of the 9 patients underwent either 2 or 3 semen retrievals.

#### Surgical sperm retrieval

In case of PVS failure, no retrograde ejaculation was looked for and TESE and MESA were offered as an alternative (n=9). The decision on performing MESA was taken by the urologist the day the surgery was performed and based upon the aspect of the epididymis (dilated or not). If MESA was performed, it was always combined with a testis biopsy in order to extract sperm (TESE) but also to look for Intratubular Germ Cell Neoplasia (histopathological analysis of the excised tissue). If MESA was not performed, only a testicular biopsy was undertaken.

For MESA, spermatozoa were aspirated from the caput epididymis and the fluid was examined under a light microscope to check for the presence of motile spermatozoa. Two patients underwent MESA and TESE and the remaining only TESE.

For TESE, two testicular specimens approximately 0.5 cm long and 5 mm thick were excised from two different locations on the same testis and minced using sterile needles in a dish containing 2 ml of culture medium (Ferticult flushing, Fertipro, Belgium). The minced tissue was incubated at 37°C under 5% CO_2_ for 30 min before checking for motile spermatozoa under an inverted microscope and spermatozoa were frozen.

#### Semen analysis

Immediately after liquefaction (30 min at 37°C), semen parameters were determined in accordance with the guidelines of the World Health Organization [24]. Briefly, concentration, progressive motility, total motility, viability and morphology were assessed. The morphology was determined using David’s classification, the French method, [25] and the viability was measured in accordance with WHO guidelines [24] using the eosin-nigrosin test [26] and as described previously by Bechoua et al. [27].

In the antegrade group, the parameters analyzed after thawing were progressive motility (PM: a+b), total motility (TM: a+b+c) and viability.

For the patient who underwent SSR, we determined only if motile spermatozoa were present after thawing (a, b or c). Therefore, because of a low sperm count, the number of motile spermatozoa was determined on several 10 μl drops of the sperm suspension covered with mineral oil.

#### Sperm cryopreservation

Sperm cryopreservation was performed as an option for fertility preservation. Sperm characteristics were evaluated before freezing and after thawing. Semen samples obtained either by antegrade ejaculation (PVS and SI) or by SSR were cryopreserved into freezing straws (Cryo Bio Systems, France) in liquid nitrogen using a commercial freezing medium (SpermFreeze, Fertipro, Belgium) according to the manufacturer’s instructions.

#### Sperm preparation for ICSI

After thawing, semen obtained by antegrade ejaculation was washed with a culture medium (Ferticult flushing, Fertipro, Belgium) and centrifuged at 600 g for 5 min at RT. The sperm pellet was re-suspended into 0.5 ml of culture medium (Ferticult flushing, Fertipro, Belgium) and loaded onto 2 puresperm density gradients (0.5 ml of 95% and 47.5%; Puresperm 100, Nidacon, Sweden) and centrifuged at 300 g for 20 min. After centrifugation, the sperm pellet was washed once with a centrifugation at 600 g for 5 min at RT with 1 ml of culture medium and used for ICSI. For testicular spermatozoa, only a wash with a centrifugation at 600 g for 5 min at RT with 1 ml of culture medium (Ferticult flushing, Fertipro, Belgium) was done.

#### ICSI cycles

ICSI was offered to 8 couples (4 patients from the antegrade ejaculation group and 4 patients from the SSR group). The female partners of the 8 SCI patients underwent ovarian stimulation. Briefly, pituitary suppression was performed with a daily injection of 0.1 mg of decapeptyl (IPSEN PHARMA, Paris, France) and ovarian stimulation was controlled with recombinant FSH. Serum estradiol levels and vaginal ultrasound measurements of follicles were used to evaluate the ovarian stimulation. hCG injection was scheduled when at least 3 follicles > 17 mm were visible. Oocyte retrieval was performed under general anesthesia, 34 to 36 hours after hCG administration. Assisted fertilization by ICSI was performed 1–3 h following oocyte collection on morphologically mature oocytes. Depending on the female age, the number of cycles, the number and quality of embryos obtained, 1, 2 or 3 embryos were transferred at either day 2 or day 3 after the oocyte retrieval. Progesterone was administered daily vaginally from the day of oocyte retrieval until the time of a negative pregnancy test or until 8 weeks of gestation. Embryo cryopreservation and frozen/thawed embryo transfer were performed as described previously [28].

#### Data from the literature

We gathered the data from the studies published in the past 10 years and focused on ICSI outcomes using fresh and/or frozen/thawed sperm obtained after PVS and SSR. A multi-criteria literature search was carried out. Filters: past 10 years, languages (French and English). Key words used for the searches: spinal cord injury – quadriplegic – paraplegic – man – men – male – males – pregnancy –pregnancies - live birth - live births – deliveries – fertility – infertility - intracytoplasmic sperm injection - in vitro fertilization – IVF – ICSI - penile vibratory stimulation - surgical sperm retrieval.

#### Statistical analysis

A Wilcoxon matched-pairs signed-ranks test was performed comparing PM (progressive motility), TM (total motility) and viability before freezing and after thawing on 8 patients for whom complete data were available (antegrade group).

## Results

### Patients’ characteristics

Overall the men’s mean age was 25.2 ± 2 5.6 years and 27.3 ± 5.7 years old in the SSR group and 23.1 ± 5.1 years old in the antegrade ejaculation group. The mean time between the day of the injury and the day of the semen retrieval was 5.7 ± 5.6 years (range=1-19 years).

The causes of SCI were road traffic accidents (63%), sports injuries (10.5%), medical conditions (10.5%), self-harm (5.3%), work- related accidents (5.3%), and bullet shots (5.3%) (Table 1).

**Table 1:** **Patients’ characteristics**

Patient no	Age at injury (Y)	Type of injury	Cause of injury	Injury level	Complete/Incomplete	Duration I-SR (Y)	BM	Previous parenthood
				**Cervical**				
1	24	Quadri	Car crash	C4-C7	na	10	-	
2	31	Quadri	Sport accident	C4-C5	C	2	+	
3	24	Quadri	Car crash	C5-C6 et T4	I	12	+	
4	15	Quadri	Sport accident	C6	C	1	+	
5	27	Quadri	Car crash	C6-C7	C	2	+	
6	38	Quadri	Car crash	C6-C7	I	7	-	2
				**Thoracic**				
				**> T10**				
7	26	Para	Bullet shot	T3-T4	C	5	+	
8	27	Para	Medical	T6	C	<1	+	
9	23	Para	Car crash	T6-T7	C	<1	+	
10	24	Para	Car crash	T7-T8	C	<1	+	
11	19	Para	Medical	T8-T9	na	14	na	1
12	22	Para	Car crash	T8-T9	na	12	+	
13	20	Para	Car crash	T10	I	2	+	
				**< T10**				
14	21	Para	Car crash	T11-T12	C	2	+	
15	33	Para	Work accident	T11-T12	C	2	na	1
16	na	Para	Car crash	T12	na	na	na	
				**Lumbar**				
17	25	Para	Suicide	L3	I	2	-	
18	33	Para	Car crash	L3-L4	na	19	+	
19	22	Para	Car crash	L2-L5	na	9	+	1

Y: year ; Duration I-SR : duration between injury and sperm retrieval ; Quadri: quadriplegia; Para: paraplegia.

BM: bladder management; “-”no bladder management used; “+”bladder management used; “na” not available (we don’t know whether or not the patient used bladder management, we don’t know if the lesion was either complete or incomplete); Medical: patient 8 (spontaneous extra-dural haematoma) and patient 11 (spinal intramedullary cavernoma); Suicide: patient 17 (self defenestration).

Previous parenthood means that parenthood has been obtained before patients became spinal cord injured.

Thirteen patients were paraplegic and 6 patients were quadriplegic. Resultant disability from these causes of spinal cord injuries were incomplete quadriplegia: 10% - complete quadriplegia: 16%, complete: paraplegia 32% - incomplete paraplegia: 10%. For 32% of the patients, the type of lesion, complete or incomplete, was not known. The patients were stratified according to the level of injury. Six patients had cervical (C4-C7), 7 high thoracic (T3-T10), 3 low thoracic (T11-T12) and 3 lumbar (L3-L5) injuries (Table 1). Thirteen of our patients had bladder management (intermittent catheterization). Four out of 19 were parents before patients became spinal cord injured.

### Semen analysis

#### Antegrade ejaculation group

This group represents antegrade ejaculation using either PVS (n=9) or antegrade ejaculation after sexual intercoiurses (n=1; patient 11). Results for number of retrieval(s), sperm parameters and number of sperm straws obtained are presented in Table 2.

**Table 2:** **Parameters of semen obtained by antegrade ejaculation before freezing and after thawing (n=10)**

Patient no	1	4	7	10	11	12	13	16	17	18	Mean	SD
Parameters before freezing												
**No of retrieval (s)**	1	1	3	1	1	2	1	2	2	1	1.5	0.7
**Volume (mL)**	5.4	0.15	0.6	0.1	1.3	0.45	3.4	3.4	0.3	2	2	1.7
**Concentration (×10** ^**6**^ **/mL)**	112	280	19	5	40	0.055	78	35.8	38	210	82	88.9
**Total sperm count (10** ^**6)**^	605	42	11	1	52	0.025	265	122	11	420	153	199.6
**PM (a+b) %**	42	20	19	0	23	drop	13	35	33	34	24	12.3
**TM (a+b+c) %**	49	29	37	3	28	drop	19	48	54	40	34	15.3
**Viability %**	52	38	50	38	62	nd	42	62	66	53	51	9.9
**Morphology %**	51	10	36	4	7	nd	31	35	27	23	25	14.6
**number of sperm straws**	19	5	11	2	5	5	12	29	10	10	11	7.6
**No of spz per straw (M)**	20	2.6	4.3	0.6	3.6	0.01	12	7.2	5.8	18.3	16.8	4.2
Parameters after thawing												
**PM (a+b) %**	10	8	3.3	drop	13	drop	1	22.5	0	14	7.7	6.0
**TM (a+b+c) %**	20	11	8	drop	21	drop	2	31	16.5	25	16.9	10.2
**Viability %**	52	38	24	38	62	nd	17	47	30	53	40.1	14.7

Antegrade ejaculation group composed by 9 patients with PVS and one patient after sexual intercourse (patient 11).

nd (not determined) which means not enough spermatozoa to perform the analysis; drop: the number of spermatozoa was determined on several 10 μl drops of sperm suspension covered with mineral oil.

PM: progressive motility; TM: total motility; morphology: French classification David % [25]; SD: standard deviation, No: number; spz: spermatozoa; M: million.

Overall, the sperm motility (PM: 24 ± 12.3% and TM: 34 ± 15.3%) and the viability (51 ± 9.9%) were altered whereas the volume, concentration, total sperm count and the morphology remained in the normal reference ranges [24]. Among these patients, only one patient (patient 16) had all the sperm parameters within the normal range. The mean number of frozen straws was 11 ± 7.6 (range: 2 to 29 straws).

A Wilcoxon matched-pairs signed-ranks test was performed comparing PM, TM and viability before freezing and after thawing (complete data for n=8). Significant differences were obtained for the 3 analyzed parameters (PM: p=0.0116; TM: p=0.0116; Viability: p=0.0487), see Table 2.

#### Surgical sperm retrieval group

TESE ± MESA yielded spermatozoa in all the 9 patients who underwent surgical procedures. The mean average of sperm count per biopsy was 1.5 ×10^6^ ± 1.1×10^6^. The mean number of spermatozoa per straw was 0.1 million (Table 3).

**Table 3:** **Parameters of spermatozoa obtained by surgical extraction (n=9)**

Patient n°	2	3	5	6	8	9	14	15	19		
										Mean	SD
**No spz/biopsy (10** ^**6**^ **)**	0.2	3.6	1.2	0.5	2	2.5	0.1	1.8	1.5	1.5	1.1
**No spz/straw (10** ^**6**^ **)**	0.02	0.21	0.03	0.04	0.11	0.23	0.01	0.13	0.05	0.1	0.1
**PM %/straw**	14	<1	0	<1	0	2	0	0	0	2.3	4.8
**TM %/straw**	30	<1	2	1	<1	13	0	<1	<1	7.7	11.0
**No of straws cryopreserved**	11	17	44	13	19	11	9	14	32	18.9	11.0
**Motile spz after thawing**	+	+	+	-	+	+	-	-	-		

No : number; Spz: spermatozoa; PM: progressive motility; TM: total motility; SD: standard deviation.

+: presence of motile spermatozoa after thawing.

-: absence of motile spermatozoa after thawing.

Patients 8 and 9 underwent MESA and TESE.

Patients 2-3-5-6-14-15-19 underwent only TESE.

Eight out of nine patients had motile spermatozoa before freezing whereas only 5 patients had remaining motile spermatozoa after thawing. In case of absence of motile spermatozoa after thawing, a hypo-osmotic swelling test was performed to assess the sperm membrane integrity before microinjection [29].

#### Pregnancy outcomes

Eight couples (4 couples in each group) underwent ICSI. The fertilization rates were 57% and 55%, the embryo cleavage rates were 90% and 93% in the antegrade ejaculation and surgical sperm retrieval groups respectively.

Five out of the eight couples achieved pregnancy. Four pregnancies were obtained after the transfer of fresh embryos (patients 1-12-6-8) and one pregnancy after a frozen/thawed cycle (patient 5). The cumulated pregnancy rates taking into account fresh and frozen embryo transfers were 50% and 75% in the antegrade ejaculation and surgical sperm retrieval groups respectively.

Among the 5 gestations, 1 twin gestation occurred (patient 8). Six healthy babies were born (4 singletons and one pair of twins) (Table 4).

**Table 4:** **Comparison of pregnancy outcomes between patients who underwent ICSI using frozen/thawed sperm, in the antegrade ejaculation group (n=4) and surgical (n=4) groups**

	Antegrade				Surgical			
Patient No	1	11	12	18	3	5	6	8
***Fresh cycles with frozen/thawed sperm***								
Duration I-SR (y)	10	na	12	19	12	2	7	<1
Care duration	1	2	1	3	1	<1	<1	1
Partner’s age(y)	22	29	32	36	35	20	28	22
No of ICSI cycle(s)	2	5	2	3	2	1	1	2
No of follicules retreived	5/10	10/12/16/18/19	6/5	6/10/16	6/6	9	14	25/18
No of injected oocyte(s)	1/8	1/6/9/11/11	2/2	5/5/6	6/5	6	9	15/15
No of zygote(s)	1/5	0/2/3/7/4	1/2	4/3/5	6/1	5	6	0/11
Fertilization rate %	100/63	0/33/33/64/18	50/100	80/60/83	100/20	83	56	0/73
No of embryos obtained	1/4	0/2/3/7/4	1/2	4/3/5	4/1	5	6	0/11
Clivage rate %	100/80	0/100/100/100/100	100/100	100/100/100	67/100	100	100	0/100
No of embryo(s) transferred	1/2	0/2/2/2/3	1/2	2/2/3	2/1	1	1	0/2
No of frozen embryo(s)	0/2	0/0/0/5/1	0/0	2/0/3	0	3	2	0/0
Pregnancy	1/0	0	0/1	0	0	0	1	1
Birth	**1**	0	**1**	0	0	0	**1**	**2**
***Cycles using cryopreserved embryo(s)***								
No of thawed embryo(s)		5/1		2/0/0		1		
No of embryo(s) transferred		1/1		1		1		
No of embryo(s) left		0		3		2		
Pregnancy		0		0		1		
Birth		0		0		**1**		

Care duration: the time between the first consultation and either the time a birth occurred or the patients decided not to pursue; na: data not available.

The numbers in bold correspond to live born baby(ies).

### Data from the literature

#### Sperm retrieved by SSR

For the 7 considered studies (see Table 5), in 3 out of the 7 studies, the origin of the sperm was known [15: frozen/thawed, 16: fresh, 18: fresh and frozen/thawed]. As for the 4 others [17, 19–21] the origin of the sperm used for ICSI was not clear. The mean pregnancy rate per couple using spermatozoa retrieved by SSR was 56%.

**Table 5:** **Studies of ICSI outcomes in SCI patients classified by the chronological order of date of publication**

Studies		Number of SCI patients	Sperm retrieval methods				ART			Pregnancy rate/ couple
Authors	Year of publication		PVS	EEJ	SSR	Other	IUI	IVF	ICSI	
***SSR***										
*Kyono et al.*	2001 [16]	1			✓				✓	100%
*Shieh et al.*	2003 [15]	10		✓	✓	1 sperm donation	✓		✓	80%
*Kazemnejad et al.*	2005 [20]	30		✓	✓	Masturbation			✓	13%
*Engin-Ulm Stün et al.*	2006 [21]	44		✓	✓	Prostatic massage			✓	EEJ: 25%
										SSR: 23.5%
										PM: 22.2%
*Kanto et al.*	2009 [18]	19			✓				✓	68%
*McGuire et al.*	2011 [17]	25		✓	✓		✓		✓	48%
*Kathiresan et al.*	2011 [19]	31	✓	✓	✓				✓	58%
										**Mean= 56%**
***PVS***										
*Pryor et al.*	2001 [22]	11	✓	✓			✓		✓	72%
*Kathiresan et al.*	2011 [19]	31	✓	✓	✓				✓	58%
										**Mean= 65%**

PVS: penile vibration stimulation; EEJ: electroejaculation; SSR: sperm surgical retrieval; IUI: intrauterine insemination; ICI: intracervical insemination; IVF: in vitro fertilization, ICSI : intracytoplasmic sperm injection ; PM: prostatic massage; No: number; nd: not determined.

#### Sperm retrieved by PVS

There are few reports on successful pregnancy rates after ICSI using spermatozoa obtained after PVS in SCI patients (2 studies, see Table 5). In these studies, semen was obtained by PVS and ICSI was performed. In these two studies, no information was given regarding the origin of the sperm when performing ICSI (fresh *vs* frozen/thawed).

## Discussion

In the present study, a small number of patients were included despite the high number of SCI patients recorded in Burgundy for the same time period. The reasons for such a discrepancy are the following: i) all the patients referred to our centre and for whom sperm retrieval failed, were not recorded in our sperm banking database, and ii) sperm banking was not systematically offered by the practitioners to their SCI patients.

Among the 19 SCI patients who preserved their spermatozoa, eight and their partners underwent an ICSI program (18 cycles) which resulted in five pregnancies (6 healthy babies were born).

In our series, the patients had a spinal cord lesion at different levels (from cervical vertebra 4 to lumbar vertebra 5). In all cases but one (for whom sperm was obtained after sexual intercourse), PVS was used as a first-line attempt to induce ejaculation. Sperm retrieval using PVS was successful in 47% of the cases (9 patients out of 19) and the patients concerned were distributed among the 3 categories (22% for cervical lesions, 56% for thoracic lesions and 22% for lumbar lesions). As for the 9 remaining patients for whom PVS failed (47% of the cases), MESE ± TESA was successfully performed. In the SSR group, the patients were also distributed among the 3 categories (44.5% for cervical lesions, 45.5 % for thoracic lesions and 11% for lumbar lesions).

The quality of sperm in the antegrade group was consistent with previous studies [30, 31], namely a low sperm motility (24 ± 12.3%) and low sperm viability (51 ± 9.9%). Among the 10 patients who had an antegrade ejaculation, only one had his sperm parameters within the normal ranges. This patient did not undergo ICSI.

Cryopreservation was performed for all the patients included in the study and ICSI performed for 8 of them. Our results on semen parameters after thawing are in agreement with what has been published previously [7] and indicate a detrimental effect of cryopreservation on semen parameters. Our results, which are satisfactory, cannot be compared in term of pregnancy rates to what has been published in the literature. Indeed, most of the studies lack the origin of the semen used for ICSI (fresh versus frozen/thawed). While cryopreservation is controversial, it could be offered to such patients in very specific situations (anxiety management about their future fertility; difficulty scheduling semen retrieval with oocyte retrieval).

Therefore, we conducted a survey in order to find out how the SCI patients who did not undergo ICSI (11 patients) felt about having their sperm cryopreserved. Two out of the 11 patients were lost to follow-up. As for the 9 remaining patients - 3 are still single, 2 have a girlfriend but no parental project and the remaining 4 did not want to answer our questionnaire. The two patients who had children before they became spinal cord injured (patients 15 and 19, both from the SSR group) had undergone an ART (in other fertility centres) which did not succeed because of a premature ovarian failure of their partner. Thereafter, the couples decided not to pursue pregnancy. When asked, the patients with no children expressed their fear about not being able to become a parent. Indeed, in SCI patients, parenthood represents an important aspect of their rehabilitation program. The PhD thesis by Espagnacq in 2008 [32] pointed out that SCI patients in couples without children at the time of the accident are more likely to separate. In addition, it seems that having no kids or the fear of not being able to have one is the main cause of separation. Therefore, for some patients, having cryopreserved semen may help them overcome this aspect of their lives.

The issue of fertility management of SCI patients in accordance with patient-centred care has been addressed and published lately [14]. The authors concluded that “prospective studies on the evolution of semen parameters, ejaculatory dysfunction, post-infectious obstructions and spermatogenesis impairments in chronic SCI patients are needed to provide robust data for evidence-based management approach for SCI patients’ fertility”. Hence, the authors suggested that sperm cryopreservation represents a preventive procedure to preserve the patients’ fertility.

The results of our study are encouraging. Cryopreservation could be performed in such patients as a preventive measure in very specific situations. We will be able to inform our patients of the chances of getting pregnant with cryopreserved sperm in case they choose this option. Making this retrospective study would have been very useful to re-evaluate our practices.

## Conclusions

Our study on the ICSI outcomes using frozen/thawed sperm in SCI patients brings new information as the literature lacks publications on this topic. In addition, although some studies do not recommend freezing sperm in SCI patients, the pregnancy rates presented in this study warrant the use of frozen/thawed sperm in selected patients.

## Abbreviations

ART: Assisted reproductive technology
EEJ: Electroejaculation
FSH: Follicle-stimulating hormone
ICSI: Intra-cytoplasmic sperm injection
IVF: *In Vitro* fertilization
hCG: Human chorionic gonadotropin
MESA: Microsurgical epididymal sperm aspiration
PVS: Penile vibratory stimulation
RT: Room temperature
SCI: Spinal cord injury
SSR: Surgical sperm retrieval
TESE: Testicular sperm extraction
WHO: World Health Organisation.
